# The Integrated Consideration of Vaccine Platforms, Adjuvants, and Delivery Routes for Successful Vaccine Development

**DOI:** 10.3390/vaccines11030695

**Published:** 2023-03-17

**Authors:** Michael Kozak, Jiafen Hu

**Affiliations:** 1 The Jake Gittlen Laboratories for Cancer Research, Pennsylvania State University College of Medicine, Hershey, PA 17033, USA; 2 Department of Pathology and Laboratory Medicine, Pennsylvania State University College of Medicine, Hershey, PA 17033, USA

**Keywords:** vaccine, adjuvant, delivery, vaccination, immune responses, innate immunity, adaptive immunity

## Abstract

Vaccines have proven to be the most cost-efficient and reasonable way to fight and exterminate virulent pathogens. Vaccines can be designed using a variety of platforms including inactivated/attenuated pathogen or subunits of it. The most recent COVID mRNA vaccines have employed nucleic acid sequences for the antigen of interest to combat the pandemic. Different vaccine platforms have been chosen for different licensed vaccines which all have shown their ability to induce durable immune responses and protection. In addition to platforms, different adjuvants have been used to strengthen the immunogenicity of vaccines. Among the delivery routes, intramuscular injection has been the most common for vaccination. In this review, we present a historical overview of the integrated consideration of vaccine platforms, adjuvants, and delivery routes in the success of vaccine development. We also discuss the advantages and limitations of each choice in the efficacy of vaccine development.

## 1. Introduction

### Necessity Is the Mother of All Inoculation

Vaccination is the principle of exposure of the body’s immune system to a biological stimulus such as killed or attenuated organism(s), subunit(s) of organism(s), or DNA/RNA encoding antigenic proteins of pathogens to generate an immune response including memory formation with minimal morbidity and mortality. Vaccines represent the single most cost-efficient and equitable way to combat and eradicate infectious diseases [1].

Immunization was understood well before vaccination; variolation, the principle of exposing uninfected individuals to the fluid of a smallpox (variola virus) pustule, can be traced in history as far back as 1000 BC to India [2]. Those variolated often had less severe smallpox infections afterwards and variolation significantly reduced mortality. Jenner knew about variolation and surmised that cowpox exposure might offer protection against smallpox. On the Origin of Vaccine Inoculation [3] was published in 1801. The etymology of the word *vaccine* provides context for the history of the field. *Vacca* meaning cow in Latin, is the root of the word *vaccinus*. Edward Jenner’s 1796 discovery that milkmaids who had experienced cowpox were largely immune to the much more dangerous smallpox [4] is the reason for the bovine root of what is, at present, arguably the greatest contribution to public health with at least two previously mortal diseases considered ‘eradicated’ [5].

To this day the smallpox vaccine contains live vaccinia virus [6]. Inactivated and live-attenuated vaccines followed the live virus vaccine discovery in the later 1800s when Pasteur took cerebrospinal fluid (CSF) homogenates of rabies-infected rabbits and sequentially desiccated the homogenates for what was later discovered to be the first titration from live-attenuated to inactivated virus preparation [7]. Vaccination against the first bacteria occurred around the same time when Sawtschenko and Sabolotny and their student ingested agar-grown, heat-killed *Vibrio cholerae* (‘virulent cholera broth’) and later remained asymptomatic after challenge. This was the first whole-killed vaccine and also the first oral vaccination developed [8]. The principle was quickly applied to *yersinia* and *Salmonella typhi*. The more powerful attenuation technique of serial cultivation of a pathogen in vitro or in inhabitable hosts came in 1927 when Calmette passed bovine tuberculosis bacteria 230 times in artificial media to obtain an attenuated strain to protect against human tuberculosis [9].

Toxoid vaccines were the next developed, for diphtheria and tetanus, in the 1920s. In the earliest forms, horses were inoculated with diphtheria and their sera was isolated and mixed with active diphtheria toxin and administered as a mixture for exposure and toxoid prophylaxis [10] to allow the immune system to develop a response without becoming overwhelmed. Microbiology had not yet identified the exact mechanism of pathogenicity, a requisite bacteriophage for lysogenesis, but the prophylactic combination was quickly titrated to better results. Presently toxins are neutralized prior to administration with an established technique for neutralization, formalin fixation [10].

Cell culture can be traced back to the first decade of the 1900s [11]. In the 1930s embryonated hens’ eggs were shown to be a viable medium for flu virus [12]. Subsequent experiments demonstrated that the virus was inactivated by formalin and that this inactivated virus was immunogenic in humans. By purification of the virus via high-speed centrifugation, an inactivated vaccine was made available in the late 1930s. The bivalent Influenza A and B vaccine would become available in the early 1940s [13]. A later discovery that the influenza virus had a segmented genome, along with the identification of neuraminidase and hemagglutinin, led to further breakthroughs as technologies were developed. The recognition that reassortment took place within and between influenza viruses led to a further challenge. When two viruses were cultivated together, in a process called co-cultivation, plaque formation allows for selection of clones. From there a clone with RNA segments from both parent viruses can be selected and reassortment can be both understood and harnessed, as it has been for the creation of the live and inactivated influenza vaccines and one of the rotavirus vaccines [14].

Out of the discovery of attenuation, scientists understood that passage through hosts could generate weakened strains of the virus, and the advent of commercial cell culture in the 1940s propelled vaccination forward with the ability to pass rapidly in various hosts in a lab space as opposed to large animal facilities. Polio, Measles, and Rubella were among the first viruses studied in this new means of generating a live, attenuated virus [9]. The principle was that those cells which grew best in media were developing mutations suited to the culture environment, which meant loss or modification of some original genes. The Salk vaccine, the poliovirus killed using formaldehyde, was approved in the 1950s but was supplanted by the Sabin oral polio vaccine, an attenuated vaccine, in the 1960s [15,16,17]. Sabin’s oral polio vaccine and the Measles, Mumps, Rubella, and Varicella vaccines (MMRV together) were developed through serial passage in vitro and selection of clones. Cell culture technology remains a standard for vaccine development and many of the vaccines used today are generated this way.

Genetically engineered subunit vaccines entered the fold in the late 20th century with the first being Hepatitis B surface antigen [18]. HBV does not replicate in cell culture and inoculation with HBV carrier sera led to hepatitis infection [19]. The fortuitous discovery that the boiled plasma of HBV carriers provided protection without causing infection led to the idea that a fragment of some viral component might be capable of generating immunity. The Hepatitis B surface antigen was purified from these boiled sera and made available. Chimpanzees were found to be the most suitable model for HBV sera production but the high cost necessitated an alternative for global vaccination [19]. The sequencing of the first gene had only happened in 1972 and Sanger’s method arrived in 1977 [20]. With these groundbreaking tools, identification of the nucleotide sequence and the construction of a plasmid containing the surface antigen allowed for the massive production of Hepatitis B surface antigen (HbSAg) in yeast in 1986 [21]; this being the first recombinant vaccine.

Virus-like particles (VLPs) were first described in the 1950s while studying Tobacco Mosaic Virus (TMV) [22] but did not reach clinical application until the 2000s. VLPs are multiprotein structures that closely recapitulate the organization and conformation of native viruses but, importantly, without the viral genome. Thus, they have the potential to yield safer and cheaper vaccine candidates. Additionally, they are far more immunogenic than subunit vaccines as they present repetitive, natively antigenic, epitopes on their surface in a more authentic conformation. Subunit vaccines, on the other hand, often encounter issues of misfolding of the targeted antigen or insufficient presentation to the immune system [23]. A handful of VLP-based vaccines are currently commercialized worldwide, notably, some hepatitis B virus vaccines and the majority of human papillomavirus vaccines [24].

Viral vector vaccines reached clinical trials in the early 2000s, these viral vectors have been genetically modified to prevent replication while retaining the antigen of interest, usually under the control of an exogenous promoter. Additionally, the antigen can be expressed on the surface of the vector particles, or inside the virion, which contributes to the induction of an immune response [1]. Adenoviruses are the most commonly studied viral vectors and they remain the focus of ongoing vaccination efforts against HIV, malaria, flu, and zika viruses. An adenovirus vector was used for a SARS-CoV-2 vaccine and a vesicular stomatitis vector (VSV)-based vaccine against Ebola (rVSV-ZEBOV) has recently been approved for use in humans [25]. Other promising vectors include a modified vaccinia Ankara (MVA) vector and heterologous viral vector vaccines (HVVV), a combination of two or more vectors encoding either the same or different antigen. HVVV has recently been used to generate a novel Ebola vaccine candidate and in malaria vaccine work [1]. All these vectors are designed to improve the immunogenicity of candidate vaccines. More innovative ways to apply these viral vectors will further help improve the efficacies of these vaccines.

The most salient vaccine technology of the 21st century is gene vaccination. Gene vaccines work by harnessing recent advances in biochemistry, molecular biology, genetics, and chemistry to deliver selected portions of the DNA or mRNA of the pathogen of interest without the need to tolerate any pathogenicity, thus reducing the morbidity of vaccination and obviating any mortality of vaccination owing to inoculum pathogenesis [26]. DNA vaccines were first developed in the 1990s. The earliest gene vaccines consisted only of DNA as plasmids, which are taken up by cells and translated into protein. The same principles can be harnessed with mRNA as the vehicle, offering significant advantages over DNA. *Gene vaccination* is not a household term but, “*mRNA vaccine*” certainly is owing to the incredible scalability and deployment of the two mRNA vaccines during the SARS-CoV-2 global pandemic.

The advent of cell culture in the early 1900s, the development of the electron microscope in the early 1930s, the discovery of the DNA structure and the central dogma in the 1950s, and sequencing technology in the 1970s have contributed significantly to the development of vaccines for numerous previously morbid and mortal pathogens [27]. Severe disease-associated viruses, bacteria, and toxoids are the targets of existing vaccines, while the first vaccination (a subunit vaccine) against a parasite, in this case *Plasmodium falciparum*, was put forth for global consideration only last year [27]. Among the known pathogens, one remains largely untouched by vaccines—fungi. Fungal vaccines remain mostly in the developmental stages.

## 2. Stimulating the Immune System by Vaccination

Vaccine development is art and science. Finding immunogenic substrates is the obvious challenge but at the molecular level, determining how to best activate only the portions on the immune system which generate long lasting protection while minimizing acute local and systemic inflammation and autoimmunity are the greater challenges. The innate immune system is largely responsible for activating the adaptive immune system to generate long-lasting immunity. The innate immune system generates most of the inflammatory and pyrogenic effects of acute infection. The adaptive immune system, once primed to recognize an antigen, is almost impossible to stop without immunosuppression which comes with its own host of complications. Thus, the art and the science of vaccine development is the titration of the right amount of the exact antigen in a precise conformation delivered to the appropriate cells. All nucleated cells and antigen-presenting cells (APCs) use major histocompatibility complex (MHC) class I to present endogenous peptides (or exogenous peptides in ‘cross presentation’) to cluster of differentiation (CD)8+ cytotoxic T Cells, so, in theory, all viruses can trigger a cytotoxic response. However, MHC Class II are only present on APCs and MHC II presents exogenous antigens to CD4+ T Cells which are essential for the activation of B Cells to plasma cells and for the class switching which dictates humoral immunity [28]. Thus, virally infected cells typically cannot generate an antibody response to the virus with which they are infected until encountering virions or nucleic acids extracellularly. While T cells and NK cells are the primary cells involved in viral clearance, harnessing the humoral immune system is key to generating long-lasting protection against nearly all pathogens.

### 2.1. Optimizing the Efficacy of Vaccines

After the identification of the target for a vaccine, the next steps involve choosing the vaccine platform, adjuvant, and delivery routes which can make or break a vaccine before it reaches the clinic. Given the example of COVID-19 vaccine development, vaccine formats including attenuated, inactivated, subunit, DNA/RNA, and adeno-associated vector based on the spike protein have been tested and some of them are still in the developmental stages [29]. For conventional vaccine development, the safety, immunogenicity, and protective efficacy are rigorously evaluated in preclinical animal models before moving to clinical trials.

### 2.2. Vaccine Platforms

Different vaccine platforms have been successfully used for licensed vaccines and all have been shown to be safe and effective for combating severe diseases for the general population (Table 1). To generate a large quantity of a vaccine in a time-effective and cost-effective way is desirable for contagious and deadly pathogens. For example, to counteract COVID-19, multiple vaccine platforms have been proposed and tested in either preclinical or clinal trials. The first vaccine made available for protecting against COVID-19 was an inactivated vaccine. Although it was timely, propagating a sufficient vaccine supply for a global population has proven problematic. The novel mRNA vaccine has been licensed for human use due to its ability of scale up to meet global needs.

#### 2.2.1. Attenuated Vaccines

Attenuation is most frequently done by repeatedly passing a pathogen through a series of hosts, the passing weakening, or attenuating, the pathogenicity. Unbeknownst to the original developers of these methods, the attenuation was due to mutations in virulence-related genes. Presently, mutations can be delivered, either randomly or in a targeted fashion [30,31]. Importantly, attenuated pathogens are still ‘live’ pathogens; they can still theoretically infect and this possibility, of reverting to a disease-causing agent, is a limitation. Specifically, the use of attenuated vaccines poses hazards to those with underlying immunodeficiencies or those who are immunosuppressed [32]. Additionally, the attenuated vaccine is temperature sensitive per the native pathogen’s preferred clime. Among the advantages of attenuated vaccines are that the technology is well-established and safe while generating a robust B and T cell immune response.

#### 2.2.2. Inactivated Vaccines

Pathogens can be treated with chemicals or conditions which inactivate their pathogenicity. Heat and irradiation are common methods for inactivation due to denaturing and destabilizing forces which results in the destruction of the DNA. The two most widely used chemicals for inactivation are formaldehyde and β-Propiolactone (BPL) [33]. An inactivated pathogen is incapable of causing the original infection or reverting to any pathogenic version and so is safe for the immunocompromised. Inactivated pathogens typically retain structural and functional molecules which can stimulate a robust B cell response and so an antibody response but because these pathogens are inactivated and cannot infect the host, they often fail to generate T cell responses and so typically require booster vaccinations [34]. Additionally, these vaccines are usually very stable and can be manufactured, stored, and transported quite easily.

#### 2.2.3. Toxoid Vaccines

Certain pathogens cause disease by secreting a toxin; examples include tetanus, diphtheria, botulinum, and cholera. Toxoid vaccine is created by generating the toxin and placing it into formaldehyde or other solution treatment to generate a toxoid by modifying particular amino acids and inducing minor conformational changes. The toxoid is physically and chemically similar to the native toxin with little to no toxicity yet by this benignant mimicry, cross-reacting antibodies are produced. To prevent future toxin interaction with the host. Toxoid vaccines generate a robust B cell and antibody response.

One of the shortcomings of toxoid vaccines is that large doses are often required to generate adequate immune response and a common problem with using larger doses is that tolerance to the antigen can occur. Thus, toxoid vaccines almost always necessitate adjuvants and booster dosing. The principal advantages of toxoid vaccines is non-pathogenicity; they cannot cause the disease they prevent. Toxoids are fixed by the modifications and there is no possibility of reversion to virulence. Additionally, toxoid vaccines are especially stable in long-term storage. Some disadvantages of toxoid vaccines are that they usually require adjuvant and booster dosing which increases the manufacturing and delivery needs. Some toxoid vaccines have a high incidence of local site reactions which are uncomfortable and frightening for many [35].

#### 2.2.4. Subunit Vaccines

Subunit vaccines, also called acellular vaccines, contain purified pieces of the pathogen of interest which have been specially selected for their ability to stimulate an immune response. They are inactivated vaccines. Since pathogens have many immunogens, subunit vaccines are very flexible in design and also allow for combination vaccinations. There are several types: subunit vaccines containing specific isolated proteins from viral or bacterial pathogens, polysaccharides from the cell walls of bacteria or other pathogen-specific particles. Additionally, enhancement much like an adjuvant is possible whereby a particularly immunogenic particle is used as ‘bait’ for the immune system and conjugated to a singularly less immunogenic substrate to enhance the immune response; an example is the conjugated pneumonia vaccine which is pneumococcal polysaccharide bound to an inactivated diphtheria toxin and a standard aluminum adjuvant to enhance the immune response [36]. Polysaccharide vaccines are engineered to stimulate B cell responses, causing specific antibody production most commonly IgM. This specificity primes the immune response and facilitates phagocytosis. The antigens, however, are T cell independent and therefore do not result in long-lasting immunity [37,38,39,40]. Conjugation has significantly improved the efficacy of subunit vaccination.

The unconjugated pneumococcal vaccine which was developed in 1977 is composed of polysaccharides from the capsule of pneumococci. The immune response is robust but transient with activation of an independent T cell response with negligible memory B cell activity. Conjugated vaccine technology developed in the 1980s [41]. It involves the conjugation of the bacterial polysaccharide to an immunogenic peptide to elicit a T cell-dependent response with B cell memory activation. The pneumococcal conjugate reached patients in 2000 [42].

The advantages of subunit vaccines are that they are generally safe for the immunocompromised and contain no live components. They are stable and scalable with well-established technologies and can be easily adapted and modified to increase immunogenicity.

The disadvantages include the time required to determine suitable antigens, antigen combinations and conjugates as well as the need for frequent boosters if unconjugated. Additionally, while the technology is well-established, the manufacturing of conjugated and adjuvated subunit vaccines is labor-intensive and time-intensive [43].

#### 2.2.5. Gene Vaccines

DNA and RNA vaccines are nucleic acid vaccines which use genetic material from the pathogen of interest to introduce an immunogenic portion of the pathogen to host cells. The delivered genetic material is used by the host machinery to generate the intended portion of the pathogen which the body’s immune system can then recognize as foreign against which it will generate an immune response. Gene vaccines generate both a B and T cell immune response. The advantages of nucleic acid vaccines are that they contain no live pathogen and so are not capable of causing the native disease, the combination of B and T cell response generates long-lasting immunity, and they can be easily modified; this can be done to increase expression of the desired antigen or to add immunogenic features. Additionally, gene vaccines offer scalable production and can be rapidly modified to accommodate changes in the pathogen. Lastly, gene vaccines offer promise beyond infectious agents since any protein can theoretically be encoded offering promise for further therapeutic development.

DNA vaccines work by introducing DNA to cells which requires processing to mRNA before protein can be generated while mRNA vaccines are post-processing and can be immediately translated. This difference is important because processing can sometimes cause unintended changes to the intended immunogen. mRNA vaccines offer numerous safety advantages over DNA vaccines. Primarily, the mRNA is post-processing and thus cellular processing cannot adulterate the intended immunogen whether intentionally or unintentionally and mRNA is rapidly broken down by cells when not used [44]. Additionally, for mRNA vaccines, interaction with the genome is nearly impossible. Prior to the SARS-CoV-2 global pandemic, no gene vaccines had been approved for use in humans and so the long-term effectiveness and side effects of this technology is an ongoing discovery. Two mRNA vaccines have been approved in the US for the SARS-CoV-2 pandemic [45]. The first DNA vaccine for humans was recently approved for use against SARS-CoV-2 [46].

### 2.3. Adjuvants

To increase the immunogenicity of vaccines and stimulate long-term protection, one of the earliest modifications to vaccine development involved the addition of adjuvants in the early 20th century [47]. Recent advances have shown that adjuvants can increase the biological half-life of vaccines, increase antigen uptake by antigen-presenting cells, and induce local inflammation and cellular recruitment [48,49]. What follows is not a comprehensive review of the field, for further reading please see [48,49,50].

#### 2.3.1. Aluminum Salts

Aluminum salts were the first adjuvants discovered in the 1930s and through trial, error, and some luck many more have been discovered [51,52]. Most modern vaccines employ adjuvant systems. Adjuvant systems are combinations of immunostimulatory molecules that are designed to improve and broaden the protection compared to classical formulations containing aluminum salts.

For many vaccines administered subcutaneously or intramuscularly, an aluminum salt (either the hydroxide or phosphate) is used. The addition of the aluminum salt facilitates a functional deposit, or depot, at the injection site which results in the sustained release of antigen over a longer period of time than simply injecting dilute antigen in to the tissue. The sustained release time allows for a greater number of immune cells involved in the adaptive immune response to encounter antigen. Additionally, aluminum adjuvants are taken up by immature dendritic cells and likely facilitate antigen processing in the lymphoid organs leading to high-affinity clones of antibody-producing B cells [53]. Despite the widespread use of aluminum salts, there is debate as to the exact immunologic mechanisms of improved immunogenicity. Studies using animal models have suggested that aluminum may activate the innate immune system via the NLRP3 inflammasome and one popular mechanism indicates that aluminum may upregulate the Th2 response leading to improved antibody formation [54]. Aluminum salts are currently used in diphtheria, tetanus, hepatitis, and HPV vaccines. The benefit is likely an improved Th2 response while there are rare systemic reactions. These are affordable and the technology is well-established.

#### 2.3.2. Other Adjuvants

Recent discoveries in human toll-like Receptor (TLR) and chemokine signaling have allowed for the development of newer adjuvant vaccines. In the last decade, several new adjuvants have been included in human vaccines, examples of these new adjuvants are AS01_B_ (in the new shingles vaccine), CpG (in the new Hep B vaccine), and MF59 (in the adjuvant flu vaccine). These adjuvant systems can be best distinguished by their formulations: emulsion, saponins, and liposomal are three of the most common [50].

MF59 is an emulsion and is currently only used in the influenza vaccine. It is an emulsion that is made up of sorbitan trioleate, polysorbate and squalene which promotes immunogenicity through the creation of a transient “immunocompetent” environment at the injection site. The mechanism is likely the generation of a chemokine gradient which acts on local innate immune cells, which in turn recruit APCs [55,56]. For the flu vaccine, MF59-adjuvated vaccines have been associated with reduced hospitalization rates and reduced risk of acute coronary syndrome compared to the nonadjuvanted influenza vaccine. MF59 has been associated with increases in myalgia, fatigue, and headache compared to the nonadjuvanted vaccine [57]. MF59 is a proprietary adjuvant system owned by Novartis.

AS01 is a liposome-based saponin adjuvant system that contains two immunostimulants: monophosphoryl lipid A (MPL) and a saponin molecule from the bark of a South American tree *Quillaja saponaria Molina.* It is currently only used in the Shingrix shingles vaccine and a malaria vaccine in development [58] and is postulated to operate by TLR4 activation by MPL to boost the innate immune system activation in the early vaccine response which results in a more robust adaptive immune response and memory formation as evidenced by an increased Th2 response [59]. The advantage of this system is a far more robust protection in seniors compared to other Herpes Zoster vaccines [57]. Adjuvantion is simple, AS01 is simply mixed with the antigen. The principal disadvantage is an increased injection site reaction and systemic discomfort, additionally, this too is a proprietary adjuvant licensed to other firms by Antigenics (Lexington, MA).

CpG oligonucleotides act as agonists of TLR9 which is a known stimulant of B cell effect and differentiation [60]. They are easily manufactured and added to vaccine formulations.

#### 2.3.3. The Outcome of Formulating with Different Adjuvants

Adjuvants often confer advantages big enough to preference one formulation over another [61,62]. For example, two HPV VLP vaccines went to market in the U.S. for the prevention of the high risk HPV 16 and 18 strains with the first in the late 2000s [61]. The most significant difference between the two formulations was the inclusion of coverage for additional strains with Gardasil. These two vaccines also differed in the choice of adjuvant. Gardasil entered the market first and selected an aluminium hydroxyphosphate sulfate adjuvant [63]. Cervarix entered later and selected adjuvant system 04 (AS04) [64] which contains two known adjuvants, 3-O-desacyl-4′-monophosphoryl lipid A (MPL) and insoluble aluminum salts [65]. Within a few years Cervarix was pulled from the U.S. market due to low demand [66]. The principle advantage of Gardasil is the inclusion of vaccination against additional strains of HPV, notably the HPV 6 and HPV 11 strains which are the most common cause of condylomata acuminata [67]. However, antibody response comparisons have shown Cervarix to be modestly superior to Gardasil for generating antibody response to HPV 16 and 18 [68,69,70]. The degree of cross-protection, that is the protection of closely-related non-vaccine subtypes of papillomaviruses, varies between the two vaccines and this is most likely a function of adjuvant while the breadth of cross-protection was broader for Cervarix which used a simpler adjuvant [68].

### 2.4. Delivery Routes

**Figure 1:**
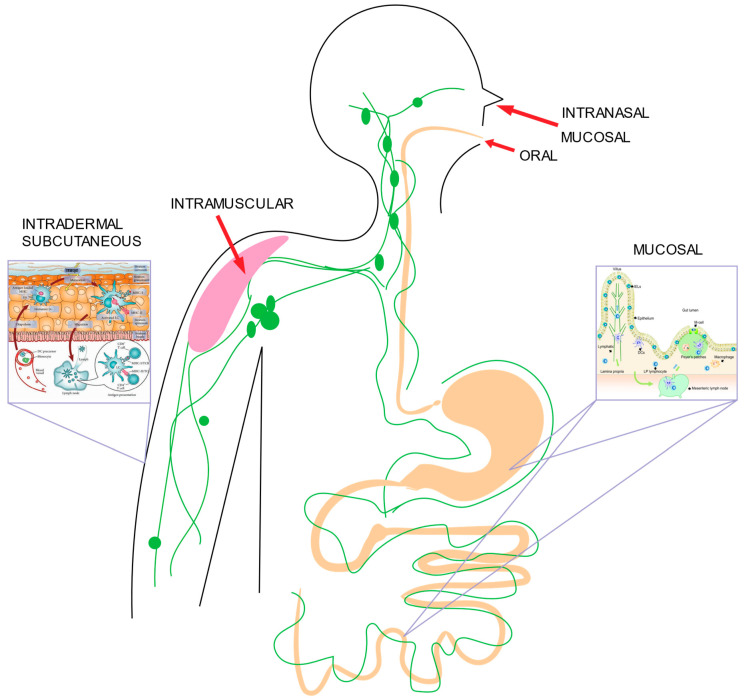
Vaccine Delivery Routes (insets (L,R): [71,72]).

Appropriate vaccine administration is the key element to ensure successful vaccination. Typically, most vaccines are administered via the subcutaneous (SC) or intramuscular (IM) routes (Figure 1). These two routes engage well-known immune surveillance systems to generate innate and adaptive immune responses while also allowing the body extended time to process the inoculum by delivering antigens and adjuvants beyond the epidermal barrier but without rapidly delivering these substances to the bloodstream which would hasten excretion.

#### 2.4.1. Mucosal Delivery

Given that the majority of pathogens enter via mucosal surfaces, a mucosal-directed vaccine is thought to generate the most robust protecting at mucosal surfaces owing to mucosa-associated lymph tissues (MALTs) and the local secretion of antibodies in to mucus at these surfaces. Mucosal delivery has several other advantages, including the absence of needles and the ability to be self-delivered. However, the numerous natural defense mechanisms of the host at mucosal surfaces, such as the acid and enzyme-rich environment of the stomach, and the mucus layer that coats all mucosal surfaces, work against successful delivery of vaccines across these surfaces which explains why to date few vaccines are delivered to mucosal surfaces. Currently, five vaccines are delivered to mucosal surfaces by two mechanisms, oral, and intranasal administration [73].

#### 2.4.2. Oral Delivery

Oral vaccines must surmount the harsh oral and gastrointestinal conditions in order to activate the immune system. This is the primary challenge to designing oral vaccines. The harsh pH and the enzymatic milieu neutralize and modify many of the existing vaccine molecules making oral administration regularly unfeasible. For vaccines which are able to succeed, there are substantial benefits, especially for vaccination against enteric illnesses because the gastrointestinal mucosa includes immune tissues. Three currently licensed oral vaccines offer protection against enteric pathogens: the oral polio virus (Sabin), a cholera vaccine, and a rotavirus vaccine (droplets placed in the mouth). The principal advantages of oral vaccination are logistical in that they are generally easier to manufacture and distribute and thus substantially more affordable than injectable vaccines and are typically more stable with longer shelf-lives making worldwide distribution easier. Additionally, many people fear needles, not just trypanophobes. Administering theses vaccines is simple and requires little training; they can also be distributed more easily than solutions.

#### 2.4.3. Intranasal Delivery

Intramuscular delivery has been shown to provide poor protection against viral replication and nasal shedding at the initial site of viral attack especially in the upper respiratory tract. In contrast, intranasal vaccines have the potential to induce sterilizing immunity against these mucosal pathogens [74]. Intranasal vaccine is administered into each nostril using a manufacturer-filled nasal sprayer; the only vaccine administered this way is one formulation of the live attenuated influenza vaccine [75]. Intranasal delivery of COVID vaccines of different platforms including virus-vectored vaccines, recombinant subunit vaccines, and live attenuated vaccines are in the development stages and potentially move to the clinical setting [76].

#### 2.4.4. Subcutaneous and Intramuscular Delivery

Subcutaneous injections are administered into the fatty tissue below the dermis and above the muscle tissue. Intramuscular (IM) injection is the most common route of vaccine delivery. IM injections are administered deep to the skin and subcutaneous into the muscle. The recommended site depends on muscle bulk and is typically based on age. The most common sites are the vastus lateralis (lateral thigh) or the deltoid (lateral shoulder) [77]. The route of administration is tailored to the vaccine, the population, and the logistics of manufacture and delivery.

Intramuscular administration optimizes the immunogenicity of most vaccinations and minimizes adverse reactions at the injection site. The muscles are highly vascularized and the vaccine can be readily mobilized and distributed to lymph tissue throughout the body. However, injecting a vaccine into the layer of subcutaneous fat, where poor vascularity may result in slow mobilization and the processing of the antigen, is a potential cause of morbidity, vaccine failure, or delayed effect [78].

Subcutaneous injection is associated with significantly lower seroconversion rates and a more rapid decay of antibody response compared to intramuscular injection. However, for certain vaccines, notably live-attenuated, this is often desirable to reduce morbidity since the antigen may take longer to reach the circulation after being deposited in fat, which is less vascular than muscle. Like a depot, this relatively decreased vascularity results in a slower and more sustained release leading to a delay in processing by macrophages and eventual presentation to the T and B cells that are involved in the adaptive immune response. One consideration is that antigens given a long exposure in fat may be denatured by enzymes if they remain for hours or days [78,79].

#### 2.4.5. Intradermal Delivery

In all injected vaccines, the innate immune response occurs at the site of injection and the adaptive immune response begins in the local lymph nodes to which the vaccine drains. Vaccine routes of administration have been compared with varying results suggesting faster or slower B or T cell responses depending on the vaccine and formulation but, generally, injected vaccines of the same formulation given by different routes yield similar results in the long term [80,81]. The Bacillus Calmette-Guérin (BCG) vaccine is a live attenuated vaccine form of *Mycobacterium bovis* used to prevent tuberculosis and other mycobacterial infections [82]. BCG is the only vaccine against tuberculosis. While not given in the United States, it is available in most other countries and is typically administered intradermally or intramuscularly. BCG vaccination very commonly results in a distinct scar at the site [83]. In general, injections are associated with distress and discomfort that is thought to result in poorer patient compliance. Injections require trained personnel for administration and are theoretically believed to additionally associated with a risk of disease transmission due to the possibility of needle-stick injuries or reuse of contaminated needles [84].

Vaccines formulated as liquids for injection are not ideal for global vaccination programs due to need for a ‘cold-chain’. Concerted efforts by researchers on alternative vaccine delivery routes have yielded a range of novel delivery devices with potential to enhance immunogenicity and stability [85].

Concerning nucleic acid vaccines, all currently licensed gene vaccines are given intramuscularly. Similar to live or attenuated viruses, DNA vaccines effectively engage both MHC-I and MHC-II pathways of intracellular and extracellular presentation inducing CD8+ and CD4+ T cells and robust B cell maturation and differentiation as well as cytotoxicity. By comparison, an antigen in soluble form, such as a recombinant protein results in a predominantly CD4+ response with resultant antibody responses although some APCs can cross-present [86]. An initial concern was that because most pathogens have three dimensional structures and motifs which are natural adjuvants, DNA vaccines would deliver inferior immunogenicity when compared to attenuated viral strains or modified bacteria. It appears that nucleic acids themselves can be adjuvants, CpG repeats being the most widely known [60]. Alternative methods of administration are being explored for efficacy and scalability using a few novel approaches, two of which are enumerated here. Comparing with intramuscular injection, both methods have shown higher immunogenicity for DNA vaccines in preclinical studies [87,88]. Gene gun delivery has been tested in clinical trials [89].

#### 2.4.6. Helium-Based Gene-Gun Delivery

In gene-gun delivery [90], plasmid DNA is precipitated onto an inert particle (generally gold beads) and forced into the cells by pressurized gas (most commonly helium). These newly transfected cells then express the instilled antigen from the plasmid leading to an immune response [91,92].

#### 2.4.7. Tattoo Gun via Electroporation

The second, and similarly mechanical, is the tattoo gun. Tattooing creates a dermal and epidermal inflammatory trauma and thus an inflammatory response [93]. Harnessing the natural inflammatory response as the optimal time to deliver plasmid has demonstrated robust immunogenicity [94].

## 3. Conclusions

The history of the polio vaccine is a perfect example of an integrated consideration of vaccine platforms (attenuated vs. inactive), adjuvants (aluminum vs. oil-in-water-based), and the routes of administration (intramuscular injection vs. oral) impacts the efficacy of a vaccine as well as the acceptance of a vaccine. Sabin’s attenuated polio vaccine has been used to immunize much of the global population because of the comparative ease and convenience of oral administration, the comparatively high level of on-site intestinal immunity that it provides in oral form, the ability of vaccination to spread to contacts of the vaccine recipients, and its low cost [95]. Inactivated polio vaccine (IPV) is believed to be the safer vaccination strategy as IPV does not mutate and is derived of pathogenicity. However, IPV is significantly more expensive than OPV. One compelling strategy to reduce the cost of IPV is to reduce the dose by adding adjuvants, however none are yet used in mass produced IPVs, work is on-going. From aluminum, used in many licensed vaccines, to newer and more experimental compounds including calcium phosphate, Freund’s complete and incomplete adjuvant, a biodegradable polymer chitosan, 1,25-dihydroxyvitamin D3, synthetic oligodeoxynucleotides with unmethylated CpG motifs (CpG-ODN), stearyl tyrosine and liposomes have been assessed with varying strengths and weaknesses [96]. A position paper written by PATH (The Program for Appropriate Technology in Health) estimated that the IPV dose, and thus price, could be reduced by as much as three quarters with an aluminum-based adjuvant, and that oil-in-water (emulsion) adjuvants could enable the IPV dose to be reduced to one tenth, highlighting the importance of adjuvants in the immunogenicity of a vaccine [97].

In the past 30 years, the sciences of vaccinology and immunology have elucidated much of the host–pathogen interaction and shed new light on the role played by antigens and adjuvants to elicit and modulate the immune response. Vaccine design has therefore become less experimental and more engineered, leading to the birth of a distinct field, ‘vaccinology’ [51]. There can be little doubt that the development of effective vaccines to combat infectious diseases is a complex multi-year and multi-stakeholder process drawing on previous research across disciplines as well as canonical principles. The current success of COVID-19 vaccine development has provided an excellent example of these integrated considerations. However, an integration of considerations for each step of vaccine development can significantly impact the final product. The choice of vaccine platform, adjuvant, and delivery route can each independently as well as collectively dictate the efficacy and success of a vaccine.

## Figures and Tables

**Table 1:** The advantages and disadvantages of different vaccines.

Vaccine	Advantages	Disadvantages	Examples
Attenuated	Preservation of native antigen; mimicking natural infection, well-established technology, robust B and T cell response	Potential to cause infection, almost all given via syringe IM, cold chain storage, not suitable for immunocompromised	Measles, Mumps, Polio (Sabin), Rotavirus, Yellow Fever, Bacillus Calmette–Guérin (BCG), Rubella, Varicella
Inactivated	Strong immune response with B cell more than T cell, waning immunity; safer than live attenuated virus—incapable of regaining pathogenicity; stable, relatively easy to scale manufacturing and distribution	Potential epitope alteration by inactivation process	Typhoid, Cholera, Hepatitis A virus, Plague, Rabies, Influenza, Polio (Salk)
Toxoid	Non-virulent, stable, and long lasting in storage	Typically not robustly immunogenic, require regular booster doses, local site reactions, given by injection	Diphtheria, Tetanus
Subunit	Readily modifiable, generally safe for immunocompromised, stable in storage and scalable in production.	Relatively less immunogenic, often require adjuvant or conjugate. Development and manufacture are typically time-consuming	Pertussis, Influenza, Streptococcus pneumoniae, Haemophilus influenzae type b
Virus Like Particles (VLPs)	Safe and well-tolerated; mimicking native virus conformation; unable to replicate; scalable and combinable with adjuvants	Relatively complicated manufacturing process; lower stability, difficult downstream processing, high production costs, and sensitivity to environmental conditions	Hepatitis B virus, Human Papillomavirus
Viral vector	Strong immune response; preservation of native antigen; mimicking natural infection	Relatively complicated manufacturing process; risk of genomic integration; response dampened by pre-existing immunity against vector	Ebola virus
DNA/RNA	Safe and well-tolerated; highly adaptable to new pathogen; native antigen expression	Requirement of low temperature storage for RNA vaccine and transportation; potential risk of RNA-induced interferon response, risk of genomic integration for DNA vaccine. Cells do not easily take up large and polar nucleic acids.	SARS-CoV-2

## Data Availability

Not applicable.
